# Mental state deterioration after switching from brand-name to generic olanzapine in an adolescent with bipolar affective disorder, autism and intellectual disability: a case study

**DOI:** 10.1186/1471-244X-13-244

**Published:** 2013-10-04

**Authors:** Rani Samuel, Azizah Attard, Marinos Kyriakopoulos

**Affiliations:** 1 National and Specialist Acorn Lodge Inpatient Children’s Unit, South London and Maudsley NHS Foundation Trust, De Crespigny Park, London SE5 8AF, UK; 2 Pharmacy, South London and Maudsley NHS Foundation Trust, London, UK; 3 Institute of Psychiatry (PO66), King’s College London, De Crespigny Park, London SE5 8AF, UK

**Keywords:** Olanzapine, Intellectual disability, Bioavailability, Generic formulation, Adolescent

## Abstract

**Background:**

The appropriateness of use of generic instead of brand-name medication remains unresolved and controversial in several areas of medicine. Some evidence suggestive of variations in bioavailability and clinical effectiveness between different formulations make policy decisions occasionally difficult. The use of generic olanzapine is a widely acceptable practice on the basis of quality, safety and efficacy data and has been adopted in several countries.

**Case presentation:**

The case of a 14 year old boy with bipolar affective disorder, autism and intellectual disability who had brand-name to generic olanzapine switch associated with rapid deterioration of his mental state is described. This clinical change was not related to any physical illness or other medication adjustment and resolved as rapidly when generic olanzapine was switched back to the brand-name formulation.

**Conclusions:**

Caution should be exercised when policy for switching from brand-name to generic psychotropic medications are made, especially when using medications off label, in extremes of age and in those patients with co-morbid complicating factors such as intellectual disability.

## Background

The appropriateness of substituting brand-name medication with generic medication policy remains unresolved and controversial in several areas of medicine. In many instances bioavailability and therapeutic efficacy vary between brand-name and generic drugs, and whether or not bioequivalence reflects clinical equivalence is still contentious [1,2]. One review highlights a few examples of clinical difficulties when switching from brand-name to generic preparations: plasma levels of phenytoin were 31% lower after a switch from a brand-name to a generic product; several controlled studies of carbamazepine showed a recurrence of convulsions after the shift to a generic formulation and sudden recurrence of seizures occurred when generic valproic acid was substituted for the brand-name product [3]. Another review highlights variations in clinical effectiveness, changes in trough plasma levels and allergies to over 25 types of psychotropic medications [4]. It has also been suggested that medications acting on the CNS, may be more prone to changes when switching from brand to generic compared to other medication categories [4]. Many hypothesise these responses to be related to differences in bioavailability between the two formulations, and statistically significant differences in pharmacokinetic variables and allergy to the inactive ingredient in favour of brand-name versus generic [2-4]. Variations in bioavailability in some patient groups could also be linked to significant differences in the way a drug is metabolised potentially affecting treatment clinical effectiveness [5].

The main reasons generic substitutions are made is the assumption of a decreased in drug costs. However, this assumption may not always be correct, in Canada for example Layton and Barbeau determined that switching patients from original to generic clozapine would lead to no cost savings if it were accompanied by an 11.2% relapse incidences caused by the change [6].

The use of generic olanzapine has been a widely acceptable practice. Araszkiewicz and colleagues highlight the safety and welcomed the use of generic olanzapine given the extensive research on relapse rates following use of brand-name, generic or both preparations in Poland where the generic preparation has been available since 2003 [7]. The US Food and Drug Administration (FDA) approved the first generic formulation of olanzapine in October 2011 [8]. Based on the review of the data on quality, safety and efficacy, the Medicine and Health care products Regulatory Agency (MHRA) also approved the use of generic olanzapine in June 2011 in the United Kingdom. To our knowledge, there is only one recent case report on adverse effects associated with switching from one preparation of olanzapine to another. In this, Goldberg described a case of akathisia following switching from brand-name to generic preparation of olanzapine in a 33 year old man with a diagnosis of bipolar affective disorder, which resolved on reverting back to the brand-name preparation [9].

## Case presentation

We present a case of a 14 year old boy with diagnoses of bipolar affective disorder, current episode mixed (ICD-10 F31.6), childhood autism (ICD-10 F84.0) and moderate mental retardation (ICD-10 F71). He had an 18-month history of gradual deterioration in his behaviour which on admission included aggression, periods of excitement interspersed with crying and head-banging, reduced need for sleep, and overactivity and was subsequently admitted to our inpatient unit. He was observed to present with disinhibition and at times displayed inappropriate behaviour towards fellow female inpatients and staff members.

His drug history includes a trial of risperidone, up to 2 mg per day which was partially successful but was associated with akathisia; a three week trial of quetiapine up to 200 mg daily was clinically ineffective and had to be prematurely discontinued due to clinical deterioration. Finally, brand-name olanzapine was added and titrated to 7.5 mg daily. In addition sodium valproate was titrated to 800 mg twice daily. This combination yielded the best improvement, in terms of mental state and functioning both at home and on the inpatient unit. At that point, after a 3-week period of sustained improvement, due to changes in our hospital budgeting pharmacy policy, generic olanzapine was given to the boy for the first time instead of the brand-preparation. This change was not apparent to the patient due to his neurodevelopmental problems. A noticeable deterioration in his mental state was observed within 2 days following the change to generic olanzapine. No other changes in medications were made. Symptoms that re-emerged included increased agitation, aggression, reduced sleep and disinhibition. This was not associated with any physical health problems, change in other medication or environmental factors. The generic olanzapine was increased to 10 mg daily with no positive effect on his mental state. Following this, the change from brand-name to generic olanzapine was hypothesised to account for the boy’s mental state deterioration. The brand-name olanzapine was restarted at 10 mg daily, with improvement in his mental state within 1 – 2 days.

## Discussion

We present a case of a 14 year boy with an acute clinical deterioration within 48 hours when changed to generic brand olanzapine followed by subsequent return to a previous stable mental state. Although Goldberg described an emergence of a new side-effect [9] we are describing a decline in clinical effectiveness. In both cases the effects subsided on reverting from generic to brand-name olanzapine.

Most generic drugs are marketed after they have passed the bioequivalence tests, set by standard licensing agencies such as the FDA. The objective of a typical bioequivalence study is to demonstrate that the test (brand-name) and reference (generic) products achieve a similar pharmacokinetic profile in plasma, serum and/or urine. Bioequivalence studies usually involve administration of test and reference drug formulations to 18–36 normal healthy subjects, but patients with a target disease may also be used [10]. Most patients are aged between 18 to 55 years of age. Children are almost never subjects of such studies.

Research in different branches of medicine raise various views regarding the use of generic preparations. For instance, Van der Meersch et al. [10], in their systematic review highlight the poor reporting of bioequivalence studies for FDA approved drugs. Wilner [11] in his survey of neurological observations of break through seizures of stable patients, whose medication had recently changed from brand-name to generic preparations, reiterate the need for careful monitoring of drugs with narrow therapeutic index. Yim [12] in their stimulation study of Area Under the Curve (AUC) of brand-name and generic preparations has highlighted the potential dangers of switching from one generic preparation to another. Kesselheim et al. [13], on the other hand in their systematic review of majority of randomized controlled trials of cardiac drugs show that despite the proof of similar bioequivalence of brand name and generic drugs there is reluctance and negativity amongst clinicians toward use of generic drugs. Richton-Hewett et al. [14] in their study highlight the medical and economic consequences on the health care system when switching from brand-name to generic preparations of oral warfarin.

## Conclusions

In conclusion, our case study adds to the breadth of case reports and knowledge that exists on this subject. It is also accepted by the authors that in this time where drug budgets and health care costs are at the forefront of policy committee members, there is indeed a need for the production and continued use of generic psychotropic medications. However, we believe caution should be exercised, when policy for switching from brand-name to generic psychotropic medications are made. This is especially so when using psychotropic medications off label, in extremes of age where medication metabolism may be affected and in those patients with co-morbid complicating factors such as intellectual disability.

### Consent

The patient’s mother has given written consent for publication of this case report after reading the manuscript.

## Competing interests

The authors declare that they have no competing interests.

## Authors’ contributions

RS produced the initial draft including interpretation of the case findings. MK and AA critically revised the draft. All authors have read and given final approval to the version to be published.

## Pre-publication history

The pre-publication history for this paper can be accessed here:

http://www.biomedcentral.com/1471-244X/13/244/prepub
